# Cesarean delivery rates in Saudi Arabia: A ten-year review

**DOI:** 10.4103/0256-4947.51773

**Published:** 2009

**Authors:** Hassan S. Ba'aqeel

**Affiliations:** From King Abdulaziz Medical City, Jeddah. National Guard Health Affairs, Saudi Arabia

## Abstract

**BACKGROUND AND OBJECTIVES:**

In view of the global increase in the rate of cesarean deliveries (CD), with the associated higher morbidity and mortality, this study was undertaken to review CD rates and some of their determinants over a ten-year period in Saudi Arabia.

**METHODS:**

Maternity data for Ministry of Health (MOH) hospitals across 14 administrative regions and other governmental hospitals in nine clusters were collected and the corresponding rates calculated using MOH yearly statistical books from 1997 to 2006. No private hospital data are reported.

**RESULTS:**

The overall CD rate significantly increased by 80.2% from 10.6% in 1997 to 19.1% in 2006. The greatest increase of 265% was in the Northern region and the least of 32.8% was in the Royal Commission Hospitals. Both vaginal breech and operative vaginal deliveries showed a significant decrease of 38% and 29%, respectively. There was a significant negative correlation between the increasing CD rate and the decreasing vaginal breech and operative vaginal deliveries rates. The volume of annual deliveries did not influence the CD rate.

**CONCLUSIONS:**

A significant increase of more than 80% in the CD rate was observed from 1997 to 2006. A national strategy to reduce the CD rate is needed and will require upgrading of the existing vital registration system. We also recommend that current national data capturing mechanisms be expanded to include private sector data and to include indications for CD.

Cesarean delivery (CD) is one of the most commonly performed surgical procedures in Saudi Arabia. As reported by the Ministry Of Health (MOH) in 2006, there were a total of 784 145 surgical procedures in all government and private hospitals, of which 86 197 were CDs (11%).^1^ Indications for CD range from well-defined medical indications to less-defined indications. In 1985, the World Health Organization suggested that CD should not exceed 15% of the total deliveries.^2^ Globally, there is an alarming increase in the CD rate. In developed countries the rate went from about 2.5% to 6% in the 1970s to about 12% to 22% in the late 1990s.^3^ In 2004, the rate in the United States reached 29.1%.^4^ Data on CD rates in developing countries are not as easily available. A recent estimate of the overall rates in developing countries is 12% based on nationally representative data from 82 countries.^5^ Annual increases in the rates of CD has been reported for some developing countries, ranging from 5% to 11%.^6^

The increase in CD rate is of concern not only because of the associated higher morbidity and mortality compared to the vaginal route, but also for the effects on subsequent pregnancies and deliveries. A World Health Organization global survey on maternal and perinatal health in Latin America, encompassing 120 facilities in 8 countries, looked at both maternal and perinatal outcome in relation to CD cesarean delivery. Even after adjustment for demographic characteristics, risk factors, general medical and pregnancy-associated complications, type and complexity of institution and proportion of referrals, there was a significant increase in severe maternal morbidity, such as postpartum hemorrhage, admission to intensive care units and hospital stay of more than 7 days. There was also a significant increase in maternal mortality. For perinatal outcome, there was a significant increase in length of stay to neonatal intensive care of more than 7 days.^7^ The MOH in Saudi Arabia annual mortality report of 2003 revealed that among a total of 37 maternal deaths, the cause of death was related to CD in three of those deaths (8%).^8^ In a cohort of 25 000 women in Scotland, the likelihood of further pregnancy following CD was significantly reduced in comparison to vaginal delivery in the index pregnancy with an adjusted odds ratio of 0.89 (95% CI, 0.82-0.96).^9^ The risk of placental abruption and placenta previa in subsequent pregnancy after a first birth by CD was reported to significantly increase by 30% and 40%, respectively, compared to first birth by the vaginal route.^10^ A relatively large population-based study of 12 944 women from England reported a 27-fold increase in the risk of CD among women who had previous CD section compared to those who had not.^11^ The aim of this study was to review available data on the rate of CD and its determinants in Saudi Arabia.

## METHODS

The main national source for CD data is the MOH. Material for this study was obtained from the Health Statistics Yearly Books covering a ten-year period from the years 1418 AH to the most recently published report for the year 1427 AH, corresponding roughly to 1997 to 2006 AD.^1^,^12^–^20^ MOH yearly statistical books report maternity data in a series of tables with running totals in two categories, representing MOH hospitals and other governmental hospitals. MOH hospitals data are reported for each of the 14 administrative regions of the country (Riyadh, Makkah, Medinah, Qaseem, Eastern, Aseer, Tabouk, Ha'il, Northern, Jazan, Najran, Al-Bahah, Al-Jouf, Al-Qurayyat). Other governmental hospitals include King Khalid University Hospital, Riyadh; King Abdulaziz University Hospital, Jeddah, King Fahad University Hospital, Khobar, Armed Forces Medical Services Hospitals (all regions), National Guard Health Affairs Hospitals (all regions), Security Forces Hospital (Riyadh only), King Faisal Specialist Hospital, Riyadh;, Royal Commission Hospitals (all regions) and ARAMCO Hospitals (all regions). Unfortunately, no private hospital data are reported. Each of the 14 administrative regions and each of the other nine governmental groups of hospitals hereafter are referred to as reporting units in this study.

MOH yearly statistical books report maternity data as total number of deliveries, total number of normal deliveries and total number of abnormal deliveries. The abnormal deliveries are broken down into ventouse deliveries, forceps deliveries, vaginal breech deliveries, cesarean deliveries and others. Indications for CD are not reported. Although stillbirth and early neonatal death data are reported, yet there was a lot of missing data from multiple reporting units for these parameters, especially neonatal death. Therefore, no attempt was made to calculate perinatal mortality rates. Relevant data were extracted from the statistical yearly books (1997-2006) for each reporting unit into a computer dataset. This dataset was used to calculate yearly CD, operative vaginal delivery and vaginal breech delivery rates. To explore the effect of the volume of deliveries on the CD rate, reporting units were arbitrarily classified into low (less than 7500 deliveries per year, 11 units), intermediate (7500-15000 deliveries per year, 4 units) and high (more than 15 000 deliveries per year, 8 units) volumes using figures for the year 2006. Descriptive and univariate analysis was performed using SPSS for Windows, version 15 (SPSS Inc., Chicago, Illinois). The Chi-square test was utilized for proportion comparisons, the Pearson correlation for assessing relationships and analysis of variance (ANOVA) for comparisons of group means. A *P* value of less than .05 was considered significant.

## RESULTS

**Figure 1:**
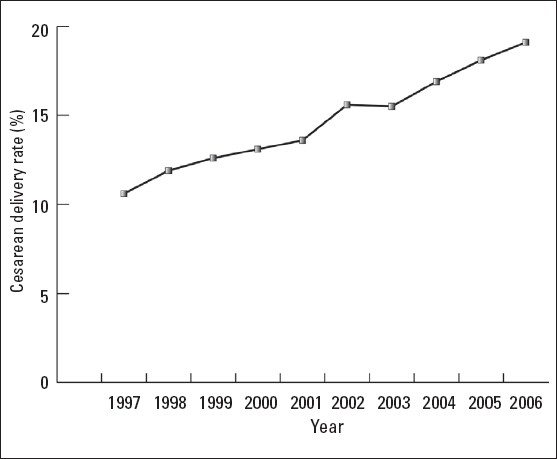
Trend of overall cesarean delivery rate (1997-2006).

**Figure 2:**
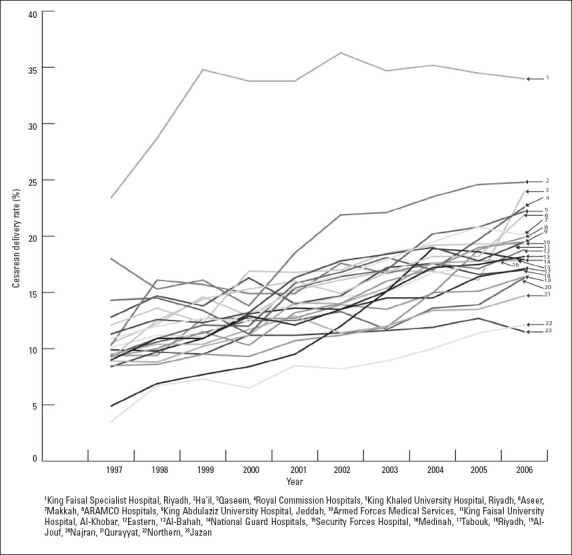
Cesarean delivery rate for each of the 23 reporting units (1997-2006).

The overall CD rate increased from 10.6% in 1997 to 19.1% in 2006 corresponding to an 80.2% overall increase (*P*<.0001). Figure 1 shows the trend in CD rate over the reporting period. All the reporting units showed a progressive increase in the CD rate through-out the reporting period (Figure 2). The amount of change in the increase in CD rate between 1997 and 2006 was quite variable among the reporting units. The percentage increase in rate was highest in the northern region (265%) and was lowest in the Royal Commission Hospitals (32.8%).

The vaginal breech delivery rate significantly dropped by 38% from 2.1% in 1997 to 1.3% in 2006 (*P*<.0001). The decrease in vaginal breech deliveries was observed across all the reporting units. The decrease in the rate of vaginal breech delivery was quite variable among reporting units. The highest decrease was observed in the Aseer region (94%) and was lowest in King Abdulaziz University Hospital in Jeddah (3%). Similarly, the rate of operative vaginal deliveries showed an overall significant 29% drop from 3.1% in 1997 to 2.2% in 2006 (*P*<.0001). Nineteen reporting units showed a decrease in operative vaginal delivery rate with the highest decrease in King Abdulaziz University Hospital in Jeddah (68.8%) and the lowest in the northern region (3%). Four reporting units showed an increase in the operative vaginal deliveries rate as follows: King Khalid University Hospital in Riyadh by 17.8%, Eastern region by 12.3%, National Guards Hospitals by 8.1% and Security Forces Hospital in Riyadh by 1.5%. There was a significant negative correlation between CD rates and vaginal breech delivery rates (r=-0.94, *P*<.0001). Similarly, there was a significant overall negative correlation between CD rates and operative vaginal delivery rates (r=-0.89, *P*=.0006).

**Figure 3:**
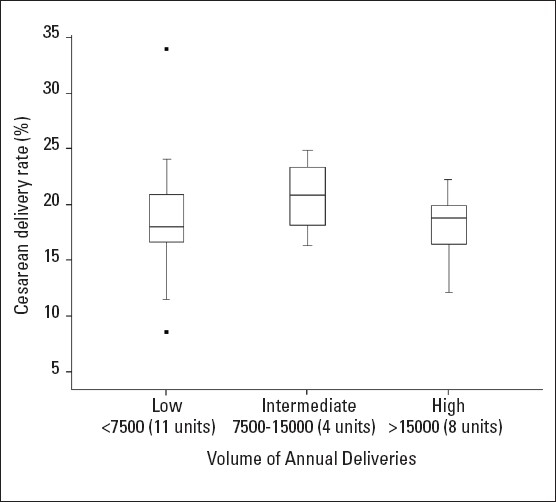
A box and whisker plot for cesarean delivery rates by high, intermediate and low annual deliveries units, for the year 2006. The central “box” represents the distance between the first and third quartiles with the median CD rate between them marked with a horizontal line. The lower end of the line below the central box “a whisker” represents the minimum CD rate, while the upper end of the other line above the box “another hisker” represents the maximum CD rate. Outlying values are shown as dots above or below the whiskers. There were no outliers for intermediate and high delivery volumes. There is an obvious overlap of the boxes and whiskers.

The total deliveries for the year 2006, whether high (more than 15 000 deliveries per year, 8 units), intermediate (7500-15 000 deliveries per year, 4 units) or low (less than 7500 deliveries per year, 11 units) did not seem to be related to the CD rate as shown in a box and whisker plot (Figure 3). The median CD rates for the high, intermediate and low were 18.8%, 20.9% and 17.9%, respectively, while the means were 18.1%, 20.7% and 18.9%, respectively. Analysis of variance revealed that the differences in the means were not statistically significant (*P*=.714). Even when the outliers were excluded the median CD rate, which is less affected by outliers, did not change, as expected, being 18.8%, 20.9% and 17.9%, respectively. The mean CD rate also changed very little, being 18.1%, 20.7% and 18.4%, respectively (*P*=.435).

## DISCUSSION

The results of this analysis of available data shows that over the period 1997 to 2006 the CD rate went from 10.6% to 19.1%, a dramatic 80.2% increase. Although data on private hospitals were not available, it is reasonable to assume that the rate of CD in the private sector would have mirrored the rate in the public sector. Some units had higher CD rates than others (King Faisal Specialist Hospital, Riyadh and Ha'il). The available data do not provide an explanation for these higher rates, which may have been due to better reporting or a different case mix. Globally, CD rates have witnessed an alarming increase over the last few years. A recent analysis of global, regional and national estimates of births by CD revealed the following means and ranges: for the World as a whole 15% (0.4-40.5%), Africa 3.5% (0.4-15.4%), Western Asia 11.7% (1.5-23.3%), Europe 19.0% (6.2-36.0%), Latin America and the Caribbean 29.2% (1.7-39.1%), North America 24.3% (22.5-24.4%) and Oceania 14.9% (4.7-21.9%).^21^ In Jordan, a report from seven military hospitals across the country revealed a rate increase from 8% for 1990-1992 to 10.9% for the 1999-2001 time periods.^22^ Another report from Jordan using data from three demographic and health surveys showed that CD increased consistently, from 8.5% in 1990, to 12.9% in 1997, to 17.8% in 2002.^23^ In Egypt data from the 1992, 1995 and 2000 demographic and health surveys revealed a 130% increase in rate from 4.6% in 1992 to 10.3% in 2000.^24^ A CD rate as high as 26.4% was reported from nine hospitals in Beirut, Lebanon.^25^ Regionally, like most developing countries, most reported CD rates reflect institutional rather than national rates. These variable sources of CD rate data coupled with a lack of national vital registration systems in most of the developing countries make meaningful in-between countries comparison unattainable.

Determinants of birth by CD include maternal age, parity, previous CD, breech presentation, multiple gestations, prolonged pregnancy and whether labor was spontaneous or induced.^3^ The volume of deliveries in a reporting unit or region has also been reported to influence the CD rate.^4^ Unfortunately, the available national data in Saudi Arabia do not report many of theses determinants. The available data allowed some insight into the influence of rates of vaginal breech deliveries, operative vaginal deliveries and volume of deliveries per reporting unit. The decrease in the vaginal breech deliveries across all reporting units was expected, especially after the publication of the Term Breech Trial (TBT).^26^ The TBT was a randomized controlled multicenter trial comparing planned CD with planned vaginal delivery of 2083 singleton fetuses in frank or complete breech presentation at 37 or more weeks of gestation. The findings suggested that planned CD reduces adverse perinatal outcomes. Of interest is the finding that the Aseer region showed the highest decrease in vaginal breech deliveries (94%). A study from Abha (in the Aseer region) maternity hospital comparing the out-come of breech delivery at term in women before and after the TBT recommendation reported a significant increase in the rate of CDs in the years following the TBT recommendation.^27^ The finding of a significant negative correlation between operative vaginal deliveries rates and CDs rates is in keeping with other studies.^28^,^29^ In contrast to Clark et al^4^ the difference in the volume of deliveries in the studied reporting units did not influence the CD rate in the current study.

The rise in the CD rate is alarming. Given the associated morbidity and mortality, it is imperative that a national strategy be considered to reduce at least some of the unnecessary CDs. The limitations and inadequacies of the current national data set preclude the development of any national strategy. It is recommended that the current national data capturing mechanisms be upgraded in terms of coverage of determinants of CD and sectors. Important parameters are missing from the current capturing tools such as indications for CD caesarean delivery. This will entail paying attention to operational definitions of the determinants, validity and reliability of the captured data, analysis and presentation for policy modification and trending analysis. Any attempt at tackling the increasing CD rate has to be data driven. Evidence-based strategies to reduce CD rates have been reported.^30^,^31^ Given the many possible reasons for deciding on CD, interventions to reduce CD rates are by necessity multifaceted. Evidence-based strategies that have shown an impact on CD rate include behavioural modification of providers, active management of labor, offering a trial of scar to women with previous CD cesarean delivery, external cephalic version for breech presentation at term, and routine induction for uncomplicated pregnancies at 41 weeks.

Possible reasons of the increase in the CD rate include improvements in the health care system in Saudi Arabia, a rising primary CD rate, provider perception of the safety of the procedure and defensive practices. However, all of these reasons are speculative.
